# Modified Hydrogels Against Phosphorus Pollution in Aqueous Environments: A Meta-Quantitative Analysis of Performance and Key Influencing Factors

**DOI:** 10.3390/gels12100859

**Published:** 2026-09-22

**Authors:** Chuang Wu, Xijun Ma, Bei Yang, Evangelos Petropoulos, Zhuo Ren, Hao Liu, Shiying He, Pengfu Hou, Tianjia Hao, Lihong Xue

**Affiliations:** 1 National & Local Joint Engineering Research Center for Mineral Salt Deep Utilization, Huai’an University, Huaian 223003, China; 2 Key Laboratory of Agricultural Environment of the Lower Reaches of the Yangtze River, Institute of Agricultural Resources and Environment, Jiangsu Academy of Agricultural Sciences, Nanjing 210014, China; 3 Key Laboratory of Integrated Regulation and Resources Development on Shallow Lakes, Ministry of Education, College of Environment, Hohai University, Nanjing 210098, China; 4 Stantec, Newcastle upon Tyne NE1 3DY, UK; vagpetrop@gmail.com; 5 Key Laboratory of Taihu Basin Water Resources Management, Ministry of Water Resources, Nanjing Hydraulic Research Institute, Nanjing 210029, China

**Keywords:** modified hydrogel, reinforced component, phosphorus pollution removal, meta-analysis

## Abstract

Excessive phosphorus accumulation drives eutrophication in aquatic ecosystems, and modified hydrogels have emerged as promising materials for phosphorus removal. However, reported removal performance and optimal operating conditions vary considerably, hindering their transition from laboratory studies to full-scale application. Here, a systematic meta-analysis was conducted to quantify the overall effectiveness of modified hydrogels and identify the major factors governing their performance. Treatment effects were quantified using the natural logarithm of the response ratio (lnRR), with more negative values indicating greater phosphorus reduction relative to the reference condition. Overall, modified hydrogels significantly reduced the phosphorus levels (pooled lnRR = −1.64, 95% CI: −1.73 to −1.55), with inorganic–inorganic modification showing the strongest effect (lnRR = −2.02, 95% CI: −2.16 to −1.88). Coexisting ions, pH, and initial phosphorus concentration were identified as the principal factors contributing to variation in removal performance. These findings provide a quantitative basis for material design and operating-condition optimization and facilitate the practical application of modified hydrogels for phosphorus pollution control. In future, standardized data reporting and long-term pilot- and field-scale validation are still needed to support widespread practical application.

## 1. Introduction

Phosphorus (P) is an essential nutrient for sustaining life. However, excessive P inputs into aquatic environments can disrupt nutrient balance and promote eutrophication. Studies have revealed that even low total phosphorus (TP) concentrations of 0.01–0.02 mg/L in water can contribute to eutrophication under susceptible environmental conditions, leading to algal blooms, loss of aquatic biodiversity, and the rapid deterioration of water quality [1,2]. Consequently, water authorities and environmental regulators worldwide have imposed increasingly stringent limits on P discharges, particularly for effluents released near drinking-water sources, sensitive water bodies, and vulnerable aquatic ecosystems [3,4]. From an engineering perspective, conventional P removal technologies in wastewater treatment systems mainly rely on enhanced biological phosphorus removal and chemical precipitation, often followed by tertiary solids separation. However, these approaches still face several limitations, including sensitivity to operating conditions, dependence on P speciation (dissolved or particulate), competition from other wastewater constituents, high chemical consumption, and difficulties in recovering P as a reusable resource, particularly when chemically generated sludge contains undesirable contaminants such as heavy metals [5,6]. These limitations have stimulated growing interest in alternative materials that can combine efficient P removal with greater resource-recovery potential and operational flexibility.

Hydrogels have attracted increasing attention as promising materials for P capture and recovery. Hydrogels are polymeric materials with three-dimensional network structures, and their high water content, tunable porosity, abundant functional groups, and chemical modifiability enable the effective adsorption of dissolved P from aqueous environments [7]. Compared with conventional P-removal methods, hydrogel-based materials offer several potential advantages, including rapid adsorption kinetics, relatively simple operation, and tunable material properties. These characteristics have stimulated increasing interest in translating hydrogel-based adsorbents from laboratory studies toward larger-scale wastewater treatment applications. Modified hydrogels refer to hydrogel matrices whose physicochemical or biological functionality is enhanced by incorporating active components, such as metal oxides/hydroxides or bioactive substances, into the three-dimensional network of base polymers including sodium alginate, chitosan, and polyacrylamide. Such modification can be achieved through physical compositing, chemical grafting, or ion loading [8,9,10]. These strategies can overcome some intrinsic limitations of unmodified hydrogels, such as insufficient active sites or weak selectivity, thereby improving their affinity and capacity for P removal [11].

However, the P-removal performance of hydrogel-based systems depends not only on material type, but also on a range of operational variables, including pH, initial P concentration, reinforcement component, hydrogel dosage, reaction time, and temperature. Consequently, substantial variation exists among the reported removal efficiencies and adsorption responses across the literature [12,13], and even similar hydrogel materials can exhibit markedly different performance when tested under different operating conditions [13]. Such variability makes direct comparison among studies difficult and limits the extent to which results obtained under specific laboratory conditions can be generalized to practical water-treatment scenarios. The dynamic nature of real wastewater and the scarcity of large-scale, long-term studies leave it unclear as to whether laboratory-scale removal performance can be reproduced under realistic conditions. Such uncertainty regarding wastewater characteristics and operating requirements may lead to inappropriate material or reagent consumption, increasing the treatment costs and potentially compromising treatment efficiency and discharge compliance. More critically, the uncertain and sometimes inconsistent effects of key operational conditions, including pH, initial P concentration, dosage, reaction time, and temperature, make it difficult to establish reliable relationships between operating conditions and P removal performance, thereby hindering the transition of modified hydrogel technologies from laboratory studies to practical applications. Despite the growing number of studies on modified hydrogels, a systematic quantitative assessment of the relative importance of these influencing factors remains lacking.

Therefore, this study aimed to quantitatively evaluate the overall P removal performance of modified hydrogels and to identify the major sources of variation in treatment effectiveness, with particular emphasis on key factors including pH, initial P concentration, reinforcement system, hydrogel dosage, reaction time, and temperature. To achieve this objective, a meta-analysis integrating random-effects models, subgroup analyses, and meta-regression was performed in R to synthesize the available evidence and quantify the contribution of different material and operational factors to P removal performance [14]. This study systematically reviewed research on modified hydrogels for P removal published over the past decade. The primary objectives were to: (i) quantify the overall effect of modified hydrogels on dissolved P removal; and (ii) evaluate the influence and relative contribution of key material and operational factors, including reinforcement system, pH, initial P concentration, hydrogel dosage, reaction time, and temperature, to variations in P removal performance. By integrating evidence across different materials and experimental conditions, this study provides a quantitative framework for understanding the sources of performance variability and identifying more favorable modification strategies and operating conditions. The findings are expected to provide useful guidance for researchers and engineering practitioners seeking to improve the design and practical application of hydrogel-based technologies for the control and potential recovery of dissolved P.

## 2. Methods

### 2.1. Data Sources and Search Strategies

This study followed a systematic literature search and screening procedure comprising four sequential stages: (i) an initial literature search within a predefined publication period; (ii) screening based on titles and abstracts; (iii) full-text assessment according to predefined eligibility criteria; and (iv) identification of the final set of studies included in the meta-analysis. The meta-analysis protocol was preregistered on INPLASY (Registration No.: INPLASY202680049; https://doi.org/10.37766/inplasy2026.8.0049). The literature search was performed in the Web of Science database using combinations of the keywords “hydrogel”, “phosphate/phosphorus”, and “removal”. Eligible studies were required to have been published between January 2016 and December 2025 and to investigate P removal using hydrogel-based materials. The lower boundary of 2016 was chosen because only a limited number of relevant studies were available before this year, while December 2025 was used as the endpoint to define a complete 10-year publication period for systematic literature screening and quantitative synthesis. A total of 386 peer-reviewed articles were retrieved and subjected to subsequent screening. To reduce the inclusion of irrelevant studies and ensure that sufficient quantitative information was available for meta-analysis, records were sequentially evaluated on the basis of their titles, abstracts, and full texts according to the following predefined inclusion and exclusion criteria:

- Titles and abstracts of the retrieved papers were required to be directly relevant to P removal using hydrogel-based materials, thereby ensuring consistency with the scope of this meta-analysis.
- Review articles and other non-original research publications were excluded.
- Eligible studies were required to report quantitative information on P removal and sufficient experimental details for data extraction, including one or more variables such as water type, pH, temperature, reaction time, coexisting ions, hydrogel dosage, initial P concentration, and P removal performance.
- Studies included in the quantitative meta-analysis were required to provide sufficient statistical information for effect-size calculation, including the sample size, mean values, and corresponding measures of variability (e.g., standard deviation) for the relevant control and treatment groups.
- The extracted data were checked for consistency, completeness, and duplication. Studies with unresolved data errors, insufficient quantitative information, or duplicated datasets were excluded from the quantitative analysis to ensure data reliability and comparability.

### 2.2. Classification Framework

To clarify the underlying causes of differences in P removal performance, the included hydrogel systems were classified according to the composition and nature of their reinforcing components. The five categories were defined as follows: (i) bio-reinforced systems, which incorporate biological components such as microorganisms, including microalgae (e.g., *Chlorella*) and anaerobic bacteria [15,16,17]; (ii) inorganic-reinforced systems, in which a single inorganic component, such as Fe-, Y-, Ce-, or Zr-based materials, provides active sites for P binding [18,19,20,21]; (iii) organic-reinforced systems, which employ organic or polymeric modifiers such as methacryloxyethyl trimethylammonium chloride, poly(methyl vinyl ether-co-maleic anhydride), and acrylamide [22,23,24]; (iv) inorganic–inorganic composite systems, which combine two or more inorganic components, including bimetallic active sites, layered double hydroxides, metal–mineral composites (e.g., clay or diatomaceous earth), and metal–carbon composites [13,25,26]; and (v) inorganic–organic composite systems, which combine inorganic active components with organic polymers, such as La-based materials coupled with polydopamine, triethylenetetramine, or polyethylenediamine, to achieve complementary or synergistic P removal [18,21,27]. This classification was used for subsequent subgroup comparisons of P removal performance among the different reinforcement strategies.

### 2.3. Statistical Analysis

The influencing factors considered in the analysis included initial hydrogel state, water type, initial P concentration, hydrogel dosage, reaction time, temperature, pH, and the presence and concentration of coexisting ions. The effectiveness of the hydrogels in removing P was assessed based on changes in dissolved P concentration. Data were extracted from the text, tables, and figures of the included studies. Data reported in the text or tables were directly transcribed, whereas data presented only in figures were digitized using WebPlotDigitizer software (v4.7), whose accuracy and reliability for quantitative data extraction have been previously validated [28]. To reduce potential digitization errors, graphical data were repeatedly extracted and cross-checked against the original figures, and inconsistent values were re-extracted before inclusion in the meta-analysis. For studies with missing standard deviations (SDs), the Bracken (1992) method implemented in the metagear package in R was used for imputation based on the coefficient of variation derived from complete cases [29]. When studies reported standard errors (SEs) instead of SDs, SEs were converted to SDs using the following equation:

(1) $$\mathrm{SD}\;=\;\sqrt{n}\times \mathrm{SE}$$

where *n* represents the number of replicates, and SE represents the standard error.

The degree of hydrogel response to P pollution removal was quantified using the response and the logarithmic response ratios, where $y_{i}$ is the effect value, *Ye* is the mean value of the treated samples in the study data, and *Yc* is the mean value of the control:

(2) $$y_{i}=\;\ln R=\;\mathrm{LN}\;\frac{Ye}{Yc}$$

The within-subject variance $v_{i}$ corresponding to the reaction ratio:

(3) $$v_{i}=\;\frac{S_{e}^{2}}{N_{e}Y_{e}^{2}}+\;\frac{S_{c}^{2}}{N_{c}Y_{c}^{2}}$$

where *Sc* and *Se* represent the standard deviations of the control and treatment groups respectively, while *Nc* and *Ne* denote the sample sizes of the control and treatment groups. *Yc* represents the average P concentration before hydrogel treatment, and *Ye* represents the average P concentration after hydrogel treatment.

A random-effects model was used to calculate the cumulative effect and confidence intervals, where:

(4) $$\mathrm{The}\;\mathrm{weight}\;\mathrm{of}\;\mathrm{individual}\;\mathrm{case}\;\mathrm{studies}:\;w_{i}^{*}=\;\frac{1}{v_{i}+\tau^{2}}$$

(5) $$\mathrm{Overall}\;\mathrm{standard}\;\mathrm{error}:\;\mathrm{SE}=\sqrt{\frac{1}{\sum_{i}^{k}w_{i}^{*}}}$$

(6) $$\mathrm{Cumulative}\;\mathrm{effect}\;\mathrm{size}:\;\bar{y}=\frac{\sum_{i=1}^{k}w_{i}^{*}y_{i}}{\sum_{i=1}^{k}w_{i}^{*}}$$

95% confidence interval for the cumulative effect size:

(7) $$95\%CI=\bar{y}\pm 1.96\times SE$$

where $\tau^{2}$ represents the estimated between-study variance in effect sizes, reflecting heterogeneity that cannot be attributed to sampling error, and *k* represents the number of effect-size estimates included in the analysis. An overall or individual effect was considered statistically significant when its 95% confidence interval did not include zero and the corresponding *p*-value was <0.05. For comparisons among different subgroups or influencing factors, statistical significance was determined based on the corresponding *p*-value, with *p* < 0.05 indicating a statistically significant difference. Accordingly, a 95% confidence interval that excluded zero indicated a statistically significant effect relative to the null value, but did not necessarily indicate a significant difference between two groups.

A subgroup meta-analysis was conducted using a mixed-effects model to assess whether categorical influencing factors were associated with differences in P removal performance. Each factor was analyzed separately, and statistical significance was determined based on the corresponding *p*-value. For continuous variables, meta-regression was used to evaluate their relationships with effect sizes and the proportion of heterogeneity explained. For the meta-regression, the total heterogeneity measure *Q_t_* was partitioned as follows:

*Q_t_* = *Q_m_* + *Q_e_* (8)

(9) $$Q_{m}=\sum_{j=1}^{p}{w_{j}(\bar{Y_{j}}-\bar{Y})}^{2}$$

(10) $$R^{2}=\frac{\tau_{RE}^{2}-\tau_{ME}^{2}}{\tau_{RE}^{2}}\;\;$$

where *Q_t_* represents the total heterogeneity, while *Q_m_* and *Q_e_* represent the heterogeneity explained by the moderator and the residual heterogeneity, respectively. A *p*-value < 0.05 for *Q_m_* indicates that the corresponding factor has a statistically significant association with the effect size. R^2^ was used to quantify the proportion of between-study heterogeneity explained by each factor. Here, $\tau_{RE}^{2}$ and $\tau_{ME}^{2}$ represent the variances among cases in the random-effects model and the mixed-effects model, respectively.

Finally, to assess potential publication bias, funnel plots were generated and Egger’s regression test was performed, with effect sizes weighted by the inverse of the variance [30,31]. A *p*-value < 0.05 for Egger’s test was considered indicative of statistically significant asymmetry. Notably, a markedly asymmetric funnel plot combined with Egger’s test may suggest publication bias—i.e., statistically significant positive results are more likely to be submitted and published, whereas non-significant or negative studies are often delayed or omitted, leading the distribution of included effect sizes to deviate from the theoretical symmetric state. Data collection and processing were performed using Microsoft Office 2024 (Microsoft, Redmond, WA, USA), while analysis and visualization were conducted using the metafor and ggplot2 packages in R version 4.5 (Jiangsu Academy of Agricultural Sciences, Nanjing, China).

## 3. Results and Discussion

### 3.1. Article Screening and Type Classification Results

As shown in the flowchart in Figure 1a, a total of 465 records were initially identified. Of these, 79 were excluded because they were published outside the predefined 10-year period. Among the remaining records, 341 were further excluded after title and abstract screening because they showed limited or no relevance to the scope of this meta-analysis. Following these two screening stages, 45 studies proceeded to full-text assessment. After a detailed evaluation of study eligibility and data completeness, 13 studies were excluded because they lacked sufficient adsorption data or statistical information, or failed to explicitly report the initial and final phosphorus concentrations or loads required for effect-size calculation. Consequently, as shown in Figure 1a, a total of 32 published studies were included in the final meta-analysis. From these eligible studies, 1460 independent observations were extracted and used to construct the meta-analytic dataset, thereby providing a relatively broad quantitative basis for the subsequent overall, subgroup, and heterogeneity analyses [11,12,13,15,16,17,18,19,20,21,22,23,24,25,26,27,32,33,34,35,36,37,38,39,40,41,42,43,44,45,46,47].

Figure 1b illustrates the geographical distribution of studies published during the selected 10-year period on P removal by modified hydrogels, including inorganic, organic, inorganic–inorganic, inorganic–organic, and bio-based systems. The period from 2016 to 2025 was selected to capture recent advances in modified hydrogel research, reflect the current development of this field, and ensure the timeliness and contemporary relevance of the analysis. Among the 32 studies included in the meta-analysis, hydrogels modified with inorganic materials were the most prevalent, with 10 studies (31.25%) focusing on this category, whereas bio-based modified hydrogels were the least represented, with only 3 studies (9.37%). The included studies were selected based on their scientific relevance, methodological suitability, and data completeness, irrespective of the authors’ nationality; geographically, the corresponding authors were distributed across 12 countries, mainly in the Asia-Pacific region and Europe. Such geographical concentration reflects strong research activity in these regions, but may also limit the representativeness of the available evidence across different environmental and application contexts. Most of the relevant studies were published between 2020 and 2025, with a particularly notable increase in publications over the past two years, indicating growing scientific interest in modified hydrogels for P removal.

However, studies focusing on the practical application of hydrogels accounted for only 12% of the total, in contrast to the large number of laboratory-scale experiments. This marked imbalance indicates that current research remains predominantly focused on small-scale experiments conducted under controlled laboratory conditions, whereas studies involving large-scale outdoor environments or real-world water treatment scenarios are still limited. This may partly reflect challenges associated with scale-up, material stability, regeneration, cost, and the limited availability of industrial application data, some of which may also be subject to proprietary or confidentiality restrictions. Insufficient practical validation not only hinders knowledge transfer and technological development, but may also contribute to a disconnect between academic research and industrial needs, thereby reducing overall innovation efficiency. Therefore, further validation under larger-scale and realistic environmental conditions is needed to facilitate the transition of modified hydrogel technologies from laboratory studies to practical P-removal applications.

### 3.2. Overall Effect, Risk of Bias, and Influence of Individual Hydrogel Materials

A meta-analysis of the P removal performance of modified hydrogels revealed a pooled effect size of −1.64 (95% CI: −1.73 to −1.55). This value indicates that modified hydrogels possess significant P removal capacity, and the fact that the 95% confidence interval lies entirely below zero statistically validates this conclusion (*p* < 0.05). Moreover, the relatively narrow confidence interval suggests that the pooled estimate is reasonably precise, indicating a consistent overall advantage of modified hydrogels for P removal across the included observations. As shown in Figure 2a, the red line represents the pooled effect size, while the blue line represents the null line (no-effect line). The degree of deviation between the two lines intuitively reflects the significance of the P removal effect [48]. The substantial distance of the pooled effect from the null line further suggests that the observed improvement is not only statistically significant, but also reflects a pronounced overall response following hydrogel modification [31]. However, this pooled effect represents an average response across different hydrogel compositions and experimental conditions, and therefore should be interpreted together with subsequent subgroup and heterogeneity analyses. Therefore, this study statistically confirms that modified hydrogel materials exhibit an overall significant enhancement in P removal from water bodies.

To assess potential publication bias, a funnel plot was constructed, and Egger’s regression test was performed. As shown in Figure 2b, the distribution of effect sizes from the included studies exhibited a certain degree of asymmetry. Egger’s test further confirmed that this asymmetry was statistically significant (*p* < 0.05), suggesting the presence of potential publication bias in the pooled analysis of dissolved P removal [30]. However, funnel-plot asymmetry cannot be attributed exclusively to publication bias, as between-study heterogeneity may also contribute to the observed pattern. This asymmetry primarily reflects several potential limitations in previous studies in this field. On the one hand, at the publication level, studies demonstrating significant removal effects are more likely to be published than those reporting weak, non-significant, or negative outcomes, resulting in an underrepresentation of studies with less favorable results. On the other hand, at the methodological level, considerable heterogeneity exists among studies in terms of hydrogel properties, synthesis pathways, and experimental conditions (such as pH and initial P concentration), which may broaden the distribution of effect sizes and further contribute to funnel-plot asymmetry [49]. Nevertheless, this study employed a random-effects model for the meta-analysis, which incorporates between-study heterogeneity into the pooled estimate and therefore provides a more conservative overall assessment when substantial variability exists among studies. The pooled results indicate that the P removal effect of the modified hydrogels was statistically significant, with the overall effect size remaining clearly below the null value [50]. Therefore, although potential publication bias may influence the estimated magnitude of the pooled effect, it does not negate the overall trend toward improved P removal by the modified hydrogels. Accordingly, the significant pooled effect supports the potential of modified hydrogels for P removal, although the magnitude of this effect should be interpreted with caution given the observed funnel-plot asymmetry and between-study heterogeneity. More complete reporting of weak, non-significant, or null results would help reduce potential reporting bias and strengthen the robustness of future meta-analytic evidence.

The analysis of 32 selected studies, involving a total of 45 modified hydrogel formulations, showed that 30 formulations achieved significant P removal, while subgroup analysis revealed significant differences in performance among the materials (*Qm* = 343.96, *p* < 0.05; Figure 2c), highlighting the important role of material composition and modification strategy in determining P removal efficiency. Among all materials, the montmorillonite–iron cross-linked alginate hydrogel developed by Das et al. (MtIA, effect size [95% CI]: −4.28 [−5.75, −2.81]) exhibited the strongest performance, achieving >99% removal at a low initial P concentration of 5 mg/L over a wide pH range of 2–10 [36]. This strong performance under low-P conditions suggests that the combined incorporation of montmorillonite and iron-based components may provide favorable P-binding characteristics, indicating potential for the treatment of low-P waters, including eutrophic surface waters, although further validation under large-scale and field conditions is required. Additionally, the kaolin–iron cross-linked alginate beads developed by Fizir et al. (Alg@K, effect size [95% CI]: −3.18 [−3.52, −2.85]) showed good removal performance at P concentrations below 50 mg/L and maintained stability over multiple regeneration cycles, demonstrating potential advantages in both P removal and reusability [40]. In contrast, some modified hydrogels exhibited comparatively limited performance. At an initial P concentration of 8 mg/L, sodium alginate hydrogels immobilizing microalgae alone (IN, effect size [95% CI]: −1.16 [−2.61, 0.30]) and bacteria alone (IB, effect size [95% CI]: −0.27 [−1.72, 1.19]) achieved removal efficiencies of 77.36% and 29.66%, respectively, whereas the hydrogel co-immobilizing microalgae and bacteria (IBN, effect size [95% CI]: −1.54 [−3.00, −0.08]) achieved a substantially higher removal efficiency of 90.44% [17], suggesting that co-immobilization may provide complementary biological functions. Furthermore, the chitosan/cationic guar gum composite hydrogel (CS/CGG, effect size [95% CI]: −0.14 [−0.67, 0.39]) showed relatively poor P removal at initial concentrations of 25 and 50 mg/L, with calculated removal efficiencies of only approximately 8.2% and 18.0%, respectively [12]. Taken together, these contrasting results indicate that hydrogel modification does not necessarily result in improved P removal; rather, removal performance depends strongly on the type and combination of functional components introduced into the hydrogel matrix. Therefore, modification strategies should be selected and optimized according to the target P concentration and specific application conditions rather than evaluated solely on the basis of whether the hydrogel has been modified.

### 3.3. Influence of Reinforcement Systems and Underlying Mechanisms

Subgroup analyses were conducted based on different reinforcement systems and materials. The materials used to modify the hydrogels were classified into five major categories. The results indicate that differences among reinforcement systems explained 5.56% of the total heterogeneity in P removal performance (*Qm* = 90.21, *p* < 0.001; Figure 3a). The effect size reflects the relative magnitude of the P removal response associated with each reinforcement strategy. According to the subgroup analysis, the magnitude of the P removal effect decreased in the following order: inorganic–inorganic systems (effect size [95% CI]: −2.02 [−2.16, −1.88]), inorganic–organic systems (−1.80 [−1.99, −1.61]), inorganic systems (−1.09 [−1.31, −0.87]), organic systems (−0.98 [−1.23, −0.74]), and biological systems (−0.90 [−1.44, −0.36]). The stronger performance of inorganic–inorganic and inorganic–organic systems suggests that combining multiple functional components may provide more diverse or complementary P-binding sites than single-component modification strategies [18,27,32,34,35,47]. In contrast, the relatively smaller effect sizes observed for organic and biological systems indicate that their P removal performance may be more dependent on specific functional groups, biological activity, and operating conditions [22,33,41,42]. These differences therefore appear to be closely associated with the distinct structural features and P-removal mechanisms of each reinforcement system.

P removal by modified hydrogels generally involves the synergistic contribution of six major mechanisms, whose relative importance varies with hydrogel composition and wastewater conditions, particularly pH and initial P concentration [25,39]. These mechanisms include: (i) electrostatic interactions, (ii) hydrogen bonding, (iii) ligand exchange, (iv) ion exchange, (v) precipitation, and (vi) bioassimilation. Electrostatic interactions enable rapid adsorption through Coulombic attraction between positively charged or protonated functional groups on the hydrogel surface and negatively charged phosphate species [51]. Hydrogen bonding, acting as a reversible non-covalent interaction, occurs between phosphate species and hydrogen-bond-donating or -accepting functional groups, such as hydroxyl, carboxyl, and amino groups, within the hydrogel matrix [52]. Ligand exchange is particularly important in metal-modified hydrogels, where phosphate replaces surface hydroxyl groups (M–OH) coordinated to metal centers and forms stable inner-sphere M–O–P complexes [53]. Ion exchange, in contrast, involves the replacement of exchangeable ions or counterions associated with the hydrogel by phosphate species, thereby promoting their retention within the material [10]. Precipitation involves the formation of poorly soluble phosphate compounds, such as LaPO_4_, through reactions between phosphate and metal ions or metal-containing active sites (e.g., La^3+^ or Ca^2+^) in the hydrogel [54,55]. Bioassimilation occurs in biologically modified hydrogels, in which immobilized microorganisms such as microalgae or bacteria take up dissolved P as an essential nutrient and incorporate it into cellular biomass [15,16,17]. Rather than operating independently, these mechanisms frequently coexist within a single modified hydrogel, and their relative contributions are governed by the type of functional component introduced, phosphate speciation, surface chemistry, and surrounding aqueous conditions. This mechanistic diversity helps explain the substantial differences in P removal performance observed among the different reinforcement systems.

Specifically, as shown in Figure 3b, inorganic–inorganic systems can remove P through multiple complementary mechanisms, including electrostatic interactions, ligand exchange, surface complexation, precipitation, Lewis acid–base interactions, and pore filling [25]. For example, La–OH sites can undergo ligand exchange with phosphate to form inner-sphere La–O–P complexes, whereas released or available La^3+^ may react with phosphate to form poorly soluble LaPO_4_ precipitates. The coexistence of these mechanisms provides multiple pathways for phosphate capture and may account for the relatively large effect size of inorganic–inorganic systems, as well as their reported high adsorption capacities, broad operational pH ranges, and potential for regeneration and reuse [11]. Inorganic–organic systems, in contrast, combine metal-based active sites (e.g., La and Zr) with organic functional groups, particularly amine-containing groups, allowing different components to perform complementary functions [18,47]. The organic phase can improve mass transfer and structural stability, while P removal proceeds through the synergistic contribution of electrostatic attraction (e.g., between protonated –NH_3_^+^ groups and negatively charged phosphate species), ligand exchange and inner-sphere complexation (e.g., formation of La–O–P bonds), and hydrogen bonding. This combination of inorganic binding sites and organic functional groups may explain why inorganic–organic systems exhibited a stronger overall P removal effect than single inorganic or organic systems. In comparison, single inorganic and organic systems rely more strongly on a narrower range of dominant interactions, such as metal–phosphate coordination or functional-group-mediated adsorption, which may make their performance more sensitive to competing ions and changes in aqueous chemistry [20,41]. Bio-modified hydrogels remove P through the combined effects of direct adsorption and biologically mediated assimilation or transformation. The hydrogel matrix can capture phosphate through functional groups such as protonated amine groups, while immobilized bacteria and microalgae contribute to P removal through coupled metabolic processes; bacteria can degrade organic matter and generate CO_2_ that supports microalgal growth, whereas microalgae assimilate dissolved P for the synthesis of cellular constituents such as nucleic acids and phospholipids. Although this bio-based pathway offers advantages in terms of sustainability and biological compatibility, its performance is more strongly dependent on microbial activity and environmental conditions than that of purely physicochemical systems. Factors such as carbon-source availability, pH, light intensity, temperature, and oxygen conditions can therefore constrain P removal efficiency [15,17]. Overall, these mechanistic differences provide a plausible explanation for the subgroup pattern observed in Figure 3a, in which systems integrating multiple complementary physicochemical mechanisms generally exhibited stronger P removal effects than systems dominated by a single interaction or by biologically constrained processes.

### 3.4. Influence of Gelatinous Forms and Coexisting Ions and Underlying Mechanisms

Based on their initial state before P removal, hydrogels can be classified into three categories: wet, vacuum-dried, and freeze-dried. Subgroup analysis indicated significant differences in P rmoval performance among the three hydrogel states (*Qm* = 14.12, *p* < 0.05; Figure 4a), with the initial hydrogel state accounting for 0.82% of the total heterogeneity. Although all three forms demonstrated significant P removal effects, wet-state hydrogels exhibited the strongest overall performance, with a pooled effect size of −1.89 (95% CI: [−2.06, −1.72]). Vacuum-dried hydrogels ranked second, with an effect size of −1.68 (95% CI: [−1.94, −1.42]), followed by freeze-dried hydrogels, which showed a relatively smaller effect size of −1.49 (95% CI: [−1.62, −1.37]). The superior performance of wet-state hydrogels may be associated with the preservation of their hydrated network structure, which can facilitate the accessibility of active sites and reduce mass-transfer resistance during P adsorption [25,35,40]. In contrast, drying processes may induce structural shrinkage, pore collapse, or partial rearrangement of functional groups, thereby limiting the accessibility of adsorption sites to varying degrees [19,34,39]. However, because hydrogel state explained only a small proportion of the overall heterogeneity, its influence on P removal appears to be secondary to other factors such as material composition and experimental conditions.

Among the included studies, 12 employed freshly prepared wet hydrogels directly for P removal. The relatively strong performance of wet hydrogels may primarily be attributed to their fully hydrated and swollen polymer networks, in which open and interconnected aqueous channels facilitate phosphate transport, reduce internal mass-transfer resistance, and improve the accessibility of adsorption sites [56]. In contrast, only four studies employed vacuum-dried hydrogels, with typical drying temperatures ranging from 40 to 60 °C. During vacuum drying, capillary forces may cause partial collapse or shrinkage of the pore structure, resulting in reduced specific surface area and porosity [57]. This, in turn, may decrease the accessibility of active sites and increase diffusion resistance, ultimately reducing adsorption performance [19]. Meanwhile, 16 studies employed freeze-dried hydrogels, with freeze-drying times primarily ranging from 12 to 24 h. Compared with conventional drying, freeze-drying can better preserve the three-dimensional network originally present in the wet hydrogel because water is removed mainly through sublimation, thereby generating a porous structure that is generally favorable for solute diffusion and adsorption [58,59,60]. However, the present subgroup analysis showed that freeze-dried hydrogels still exhibited a smaller overall P removal effect than wet hydrogels, indicating that a highly porous dried structure does not necessarily translate directly into superior adsorption performance. Rehydration behavior, recovery of the swollen network, accessibility of functional sites, and changes in surface chemistry during drying may also influence the subsequent P removal process. Taken together, these observations suggest that the initial physical state of hydrogels can affect P removal by altering network hydration, pore accessibility, and mass-transfer behavior; however, given that hydrogel state explained only 0.82% of the total heterogeneity, its contribution appears to be relatively limited compared with material composition and other experimental factors. Therefore, the hydration state and drying method of hydrogels should be considered alongside their chemical composition and operating conditions when optimizing materials for practical P removal, particularly in field applications such as agricultural runoff treatment and farmland drainage ditches.

A subgroup analysis was also conducted to evaluate the effects of different coexisting ions on P removal by modified hydrogels. The results showed statistically significant differences among the tested ions (*Qm* = 34.05, *p* < 0.05; Figure 4b), with coexisting ions accounting for 9.03% of the total heterogeneity. Among these ions, bicarbonate (HCO_3_^−^) exhibited the most pronounced inhibitory effect, with interference observed in 26% of the tested materials. This inhibition can be attributed to competition between HCO_3_^−^/carbonate species and phosphate for reactive adsorption sites, particularly metal-based active sites. For example, the Y-containing hydrogels PC-Y^3+^ (without alkaline treatment) and PC-Y-OH (after alkaline treatment), as well as lanthanum-functionalized nanocellulose/sodium alginate-based hydrogels, showed reduced P adsorption in the presence of HCO_3_^−^ [20,47]. For La-containing materials, carbonate species can interact strongly with La-based active sites and may form La-containing carbonate phases, thereby reducing the availability of these sites for phosphate binding. HCO_3_^−^ may also compete with phosphate for positively charged adsorption sites under conditions where surface functional groups are protonated, further suppressing P adsorption [61]. In contrast, Ca^2+^ may promote P removal because it can interact with phosphate and facilitate the formation of poorly soluble Ca–P species, thereby providing an additional removal pathway [20]. The influence of coexisting ions also depends strongly on the chemical form of the active metal sites. After alkaline treatment, Y in PC-Y-OH exists mainly in the form of Y(OH)_3_, which binds phosphate through ligand exchange and inner-sphere complexation, contributing to its relatively high selectivity in the presence of competing ions [20]. Most other common coexisting anions, including Cl^−^, SO_4_^2−^, and NO_3_^−^, generally exhibited weaker interference than HCO_3_^−^, which may be associated with their relatively weaker competition for metal-based active sites under the tested conditions. In some cases, increased ionic strength may also compress the electrical double layer and reduce electrostatic repulsion between phosphate species and the hydrogel surface, thereby facilitating phosphate transport toward adsorption sites [11,25]. Overall, these results demonstrate that the effects of coexisting ions are ion-specific rather than uniformly inhibitory: ions capable of strongly competing for or altering metal active sites, particularly HCO_3_^−^/carbonate species, can suppress P removal, whereas ions such as Ca^2+^ may contribute positively through additional precipitation or binding pathways. This also explains why coexisting ions accounted for a relatively substantial proportion (9.03%) of the heterogeneity in P removal performance.

### 3.5. Influence of Other External Factors and Comparison of Influencing Factors

The meta-analysis revealed significant residual heterogeneity (*p* < 0.0001 for *Q*t) across the datasets for the five types of modified hydrogels under the random-effects model, indicating that additional factors beyond hydrogel type contributed to the observed variation in P removal performance. Therefore, meta-regression analyses were conducted to examine several commonly reported experimental variables as potential sources of heterogeneity. As shown in Figure 5a, effect sizes became less negative with increasing pH, corresponding to a gradual weakening of P removal performance. The continuous-variable meta-regression showed that solution pH explained 3.66% of the between-study heterogeneity (*Qm* = 40.12, *p* < 0.0001), confirming pH as a statistically significant moderator. This relationship may reflect changes in phosphate speciation, hydrogel surface charge, and the protonation state of functional groups as pH increases, which can alter electrostatic attraction and the availability of active binding sites [18,46]. As shown in Figure 5b, the effect size similarly became less negative as the initial dissolved P concentration increased, indicating a decline in removal efficiency. This trend may result from the progressive saturation of available adsorption sites at higher P concentrations, causing the proportion of P removed from solution to decrease. Initial P concentration explained 3.45% of the between-study heterogeneity (*Qm* = 53.01, *p* < 0.0001), demonstrating that it was also an important moderator of P removal performance [33,38]. In contrast, as shown in Figure 5c, effect sizes became more negative with increasing treatment time, suggesting improved P removal with prolonged contact. However, treatment time explained only 0.33% of the between-study heterogeneity (*Qm* = 5.31, *p* < 0.10), indicating a comparatively weak contribution relative to pH and initial P concentration. Moreover, most treatment durations in the included studies were within 1800 min (30 h), suggesting that a large proportion of P removal can generally be achieved within this time range, and that excessively extending the treatment period may provide limited additional benefit under the tested conditions. As shown in Figure 5d, increasing hydrogel dosage was associated with more negative effect sizes and improved P removal, with most studies evaluating dosages within the range of 0–4 g/L. The improvement is likely related to the greater number of available adsorption sites provided at higher hydrogel dosages. Dosage explained 2.00% of the between-study heterogeneity (*Qm* = 29.19, *p* < 0.0001), indicating a statistically significant but relatively modest contribution [11,25]. Finally, as shown in Figure 5e, as temperature increased, the effect size showed a more negative trend, accompanied by improved P removal performance. The experimental temperatures reported in the included studies ranged from 10 to 65 °C, although most experiments were concentrated within 20–35 °C, which represents a typical operating range under ambient conditions. Temperature explained only 1.12% of the between-study heterogeneity (*Qm* = 13.19, *p* < 0.10), suggesting that its influence was relatively limited within the investigated range. However, data at the lower (10–20 °C) and higher (35–65 °C) ends of the temperature range were comparatively limited, and the effects of these less frequently studied temperature conditions remain insufficiently characterized [18,40]. Overall, among the five continuous variables examined, pH and initial P concentration explained the largest proportions of heterogeneity, followed by hydrogel dosage, whereas temperature and treatment time contributed comparatively little. These results indicate that solution chemistry and pollutant loading are likely more important determinants of P removal variability than simply extending treatment duration or moderately changing the operating temperature.

A comparison based on the proportion of between-study heterogeneity explained (R^2^) by the various influencing factors indicates that coexisting ions were the most important source of variability in hydrogel P removal performance, accounting for 9.03% of the between-study heterogeneity. Specifically, Ca^2+^ may promote P removal by providing additional precipitation or binding pathways, whereas HCO_3_^−^ may inhibit P removal by competing for active adsorption sites, particularly metal-based sites [20,25]. These contrasting ion-specific effects increase the variability in P removal responses under different experimental conditions, thereby contributing substantially to the heterogeneity associated with coexisting ions. Reinforcement systems ranked as the second most important factor, explaining 5.56% of the between-study heterogeneity. Inorganic–inorganic and inorganic–organic composite systems generally exhibited stronger P removal effects, possibly because the combination of multiple functional components provides complementary adsorption and reaction sites, resulting in better P removal performance than many single-component systems. In contrast, treatment time and the initial physical state of the hydrogel explained only 0.33% and 0.82% of the between-study heterogeneity, respectively, indicating relatively limited contributions to the overall variability in P removal performance. Although different drying methods can alter pore structure, network hydration, and the accessibility of active sites, the low R^2^ associated with hydrogel state suggests that these physical differences exert a weaker influence than solution chemistry and material composition. Overall, the comparison of R^2^ values further indicates that variation in P removal performance among the modified hydrogels is more strongly associated with the effects of coexisting ions and reinforcement-system composition, whereas treatment time and the initial physical state of the hydrogel make relatively minor contributions.

## 4. Conclusions

This meta-analysis synthesizes hydrogel-based studies published over the past decade and provides a quantitative basis for material selection and optimization of P removal from aqueous environments. Modification strategy emerged as a key moderator, with subgroup comparisons favoring inorganic–inorganic systems and wet-state hydrogels. Among the environmental and operational factors, explanatory importance decreased in the order of coexisting ions, pH, initial P concentration, hydrogel dosage, temperature, hydrogel morphology, and reaction time. HCO_3_^−^ exhibited the strongest inhibition among the coexisting ions examined. These findings identify promising material categories and key conditions for further optimization.

Nevertheless, cross-study differences in experimental conditions and possible confounding among moderators may affect the interpretation of pooled estimates. Future adsorption studies should therefore standardize experimental conditions to improve the comparability and reproducibility of results across studies. Further research should prioritize continuous-flow experiments using real wastewater and repeated regeneration tests to assess long-term stability, degradation, metal leaching, and service life. Evaluating treatment costs and the recovery and reuse of captured P would further support the translation of these findings into practical technologies for P pollution control and sustainable resource recovery.

## Figures and Tables

**Figure 1:**
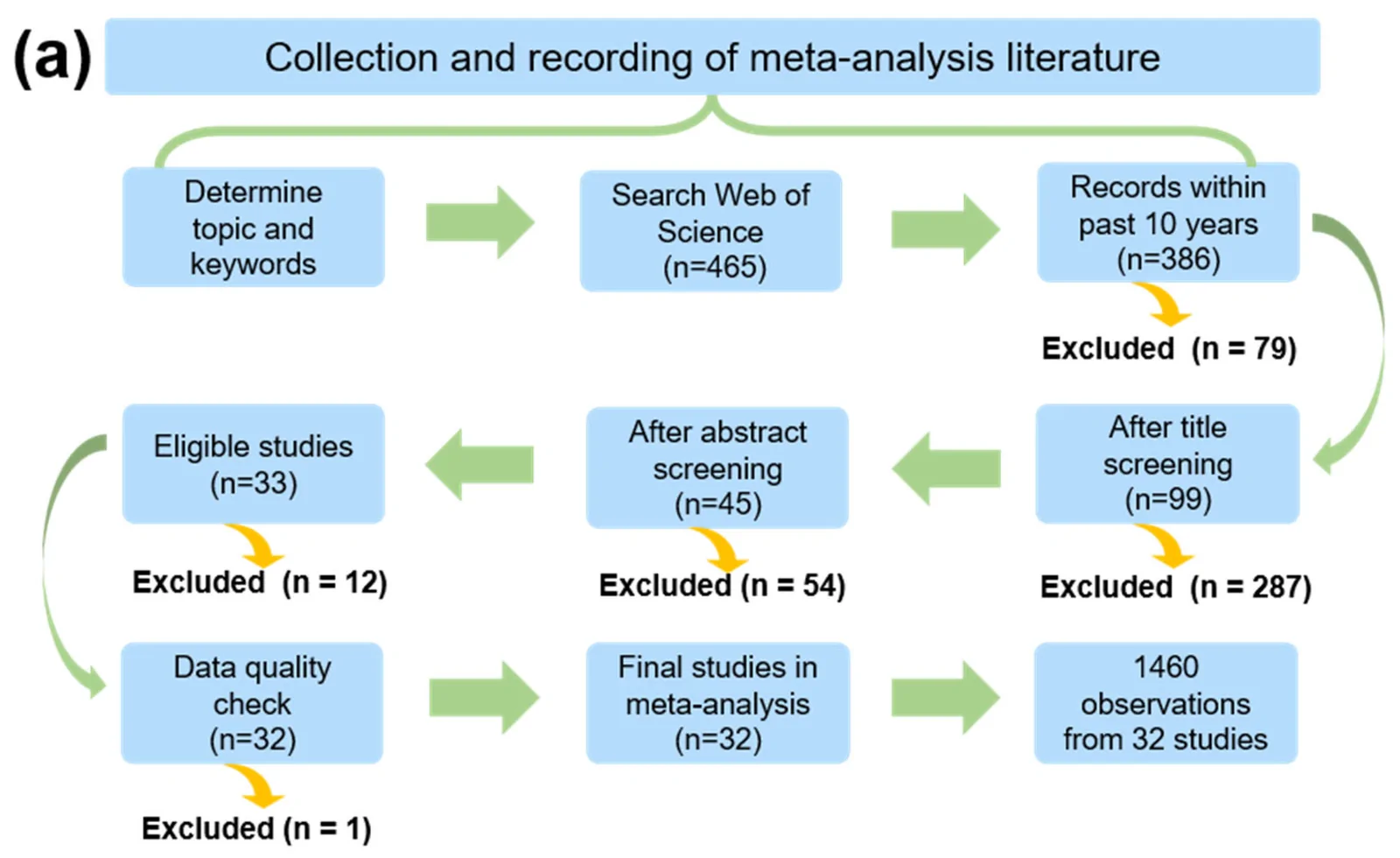

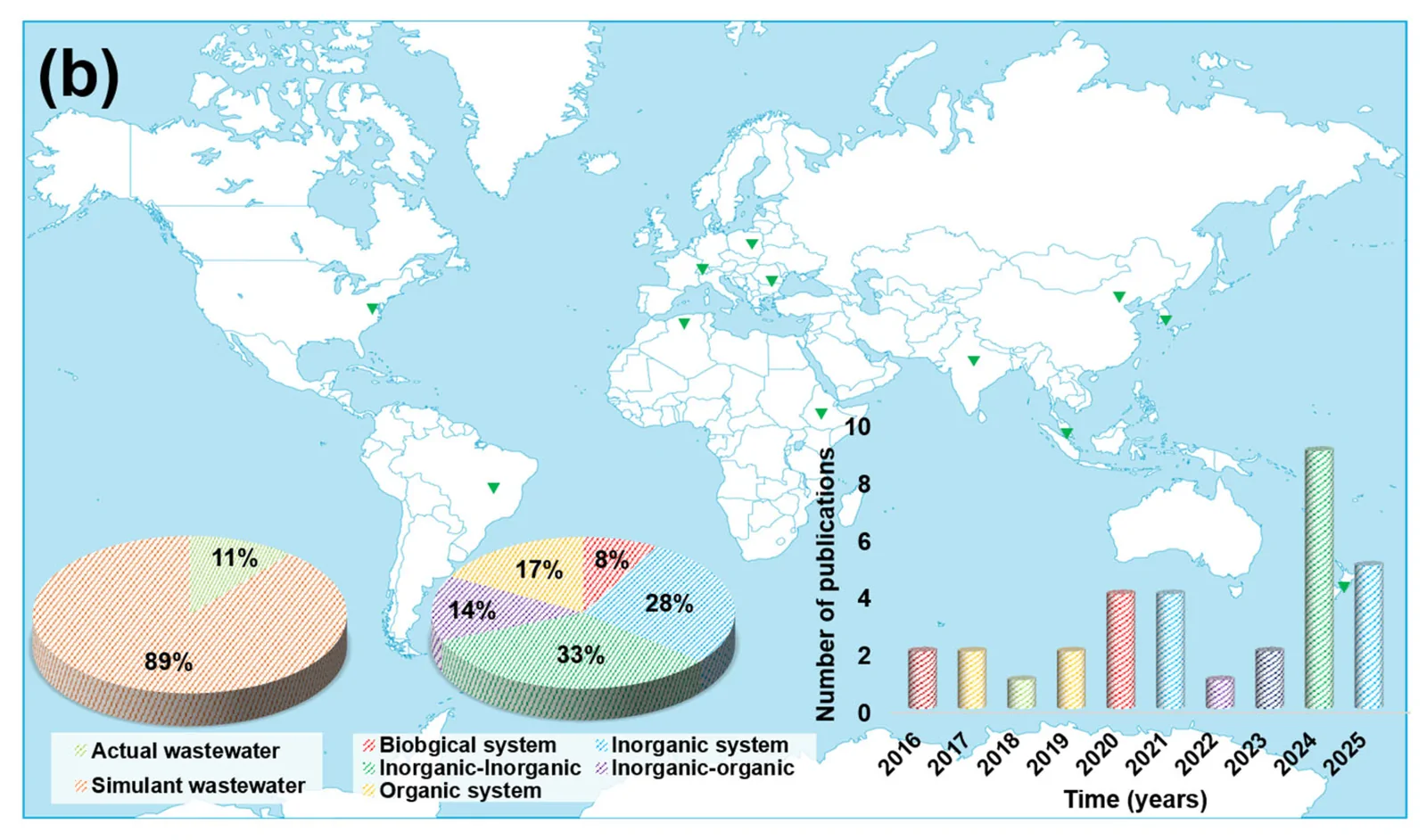
(**a**) PRISMA flowchart depicting the study inclusion and exclusion process. (**b**) Temporal trends, hydrogel enhancement systems, and geographical distribution of the eligible literature over the past decade.

**Figure 2:**
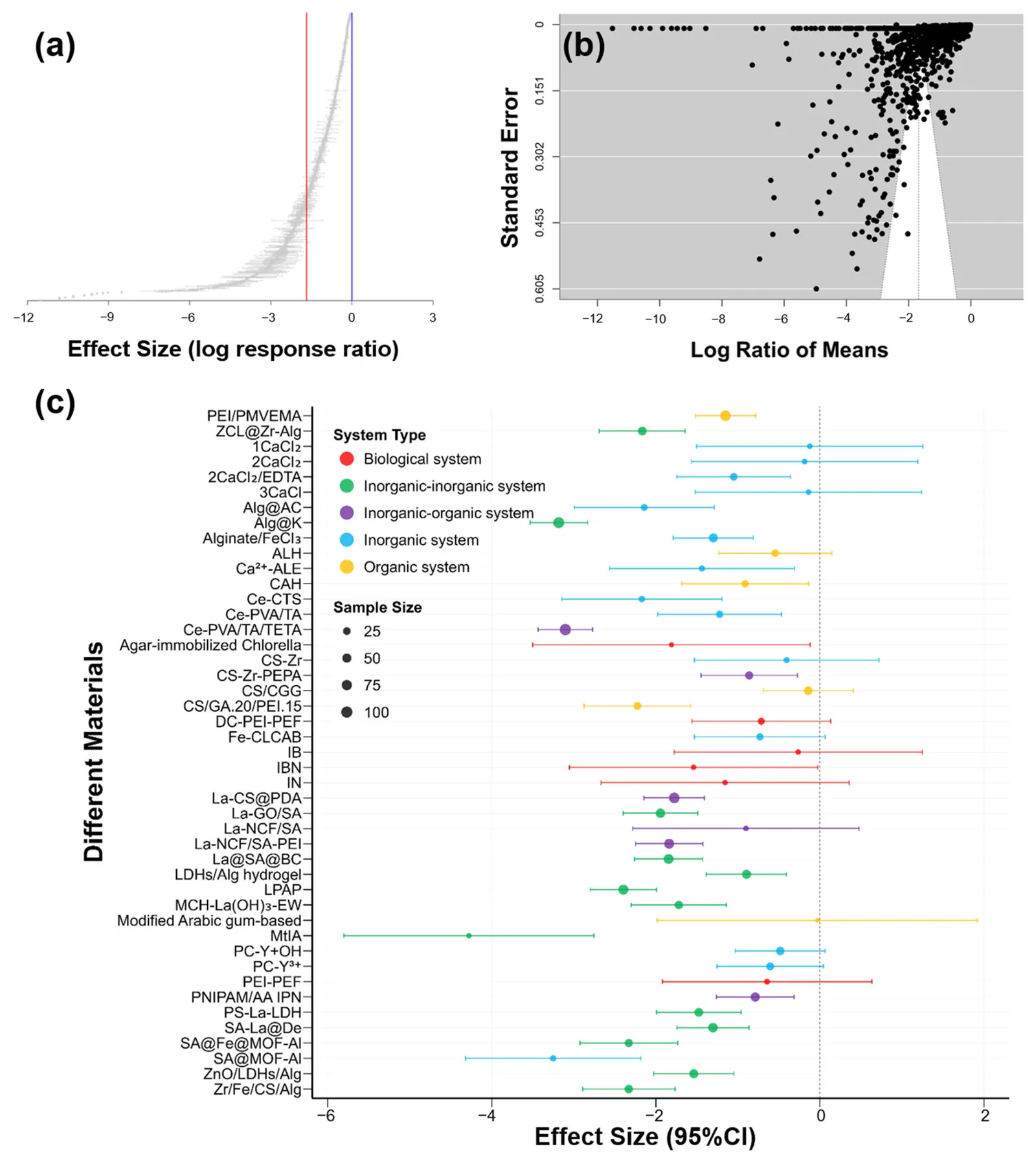
(**a**) Logarithmic response ratio, (**b**) publication bias plot of P removal by modified hydrogels, and (**c**) forest plot of P removal by different subtypes of modified hydrogels.

**Figure 3:**
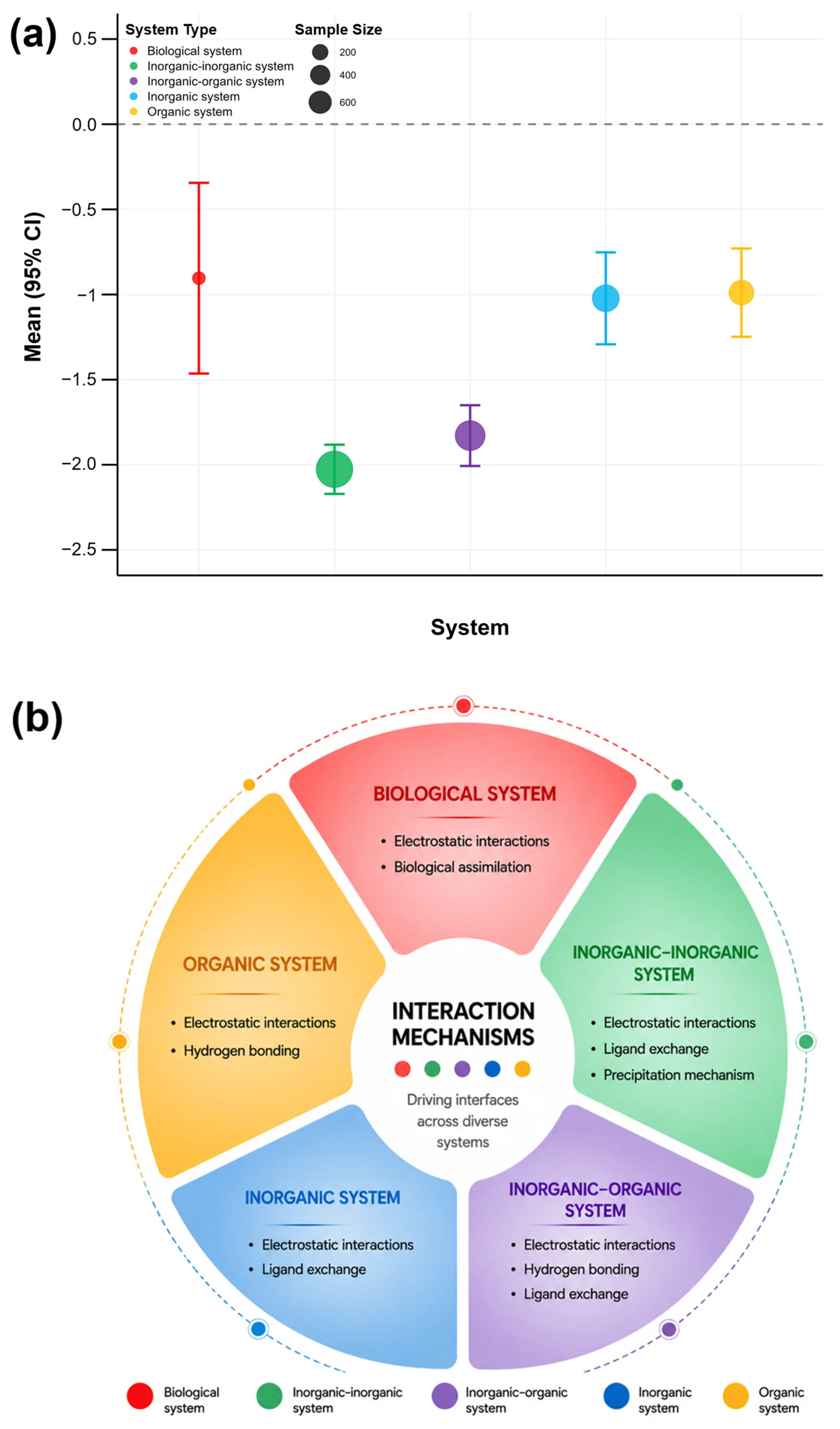
(**a**) Forest plot of P removal by enhanced systems classified by subgroup type (the position, size and error bars of the circles represent the effect value, sample size and 95% CI). (**b**) Mechanisms of action for P removal in different composition systems.

**Figure 4:**
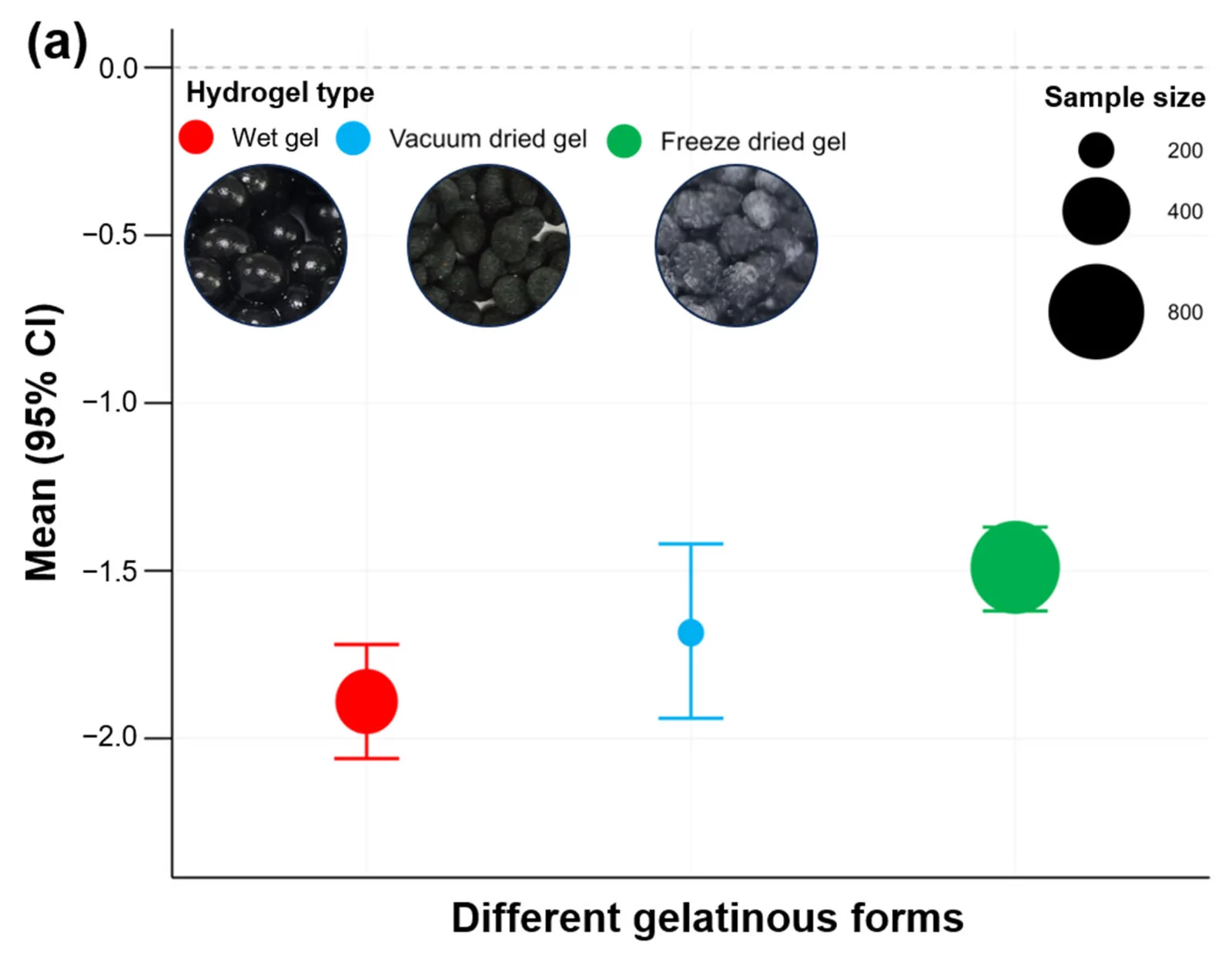

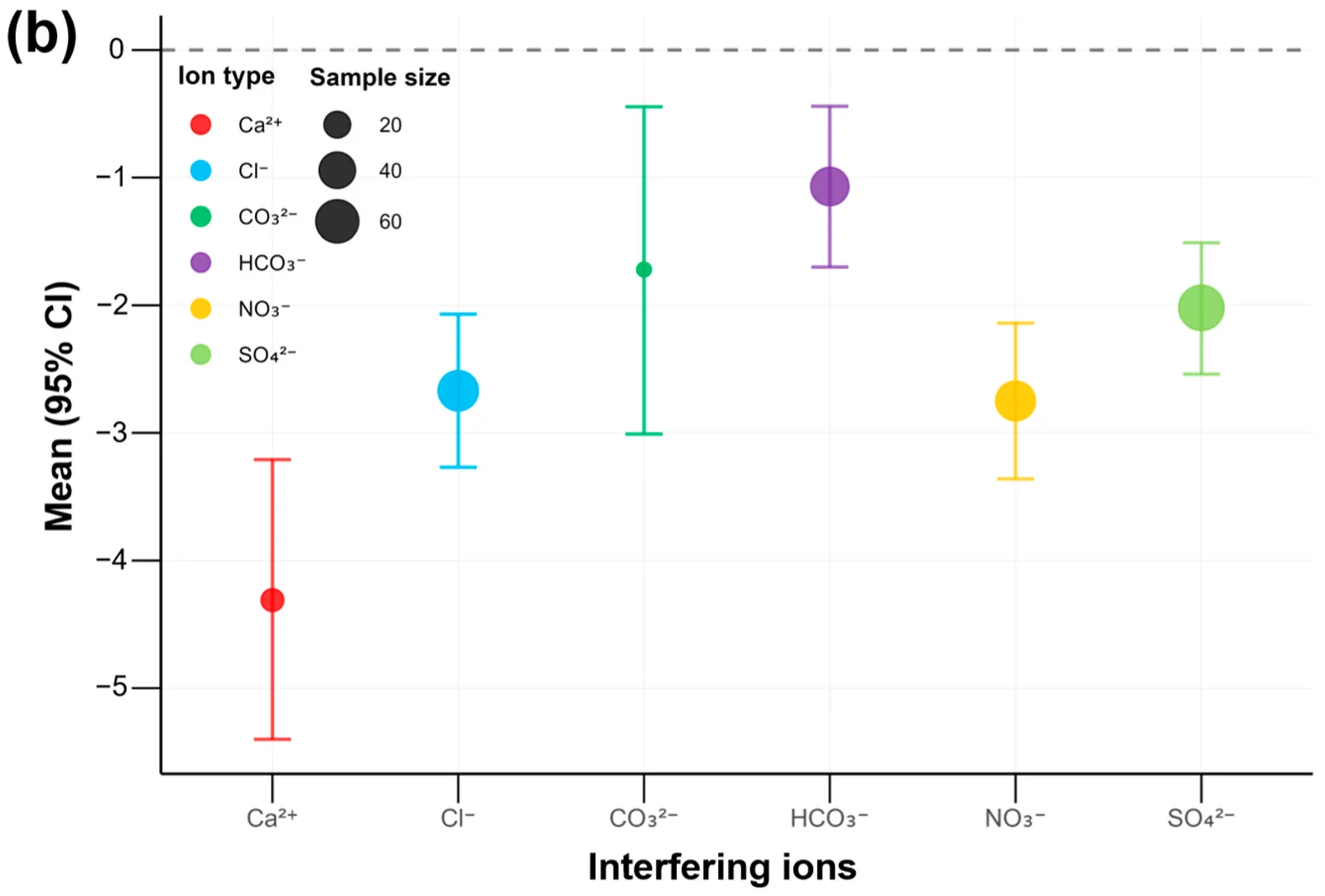
Forest map of P removal: (**a**) by different gel morphological subgroup types; (**b**) by different subgroups of interfering ions.

**Figure 5:**
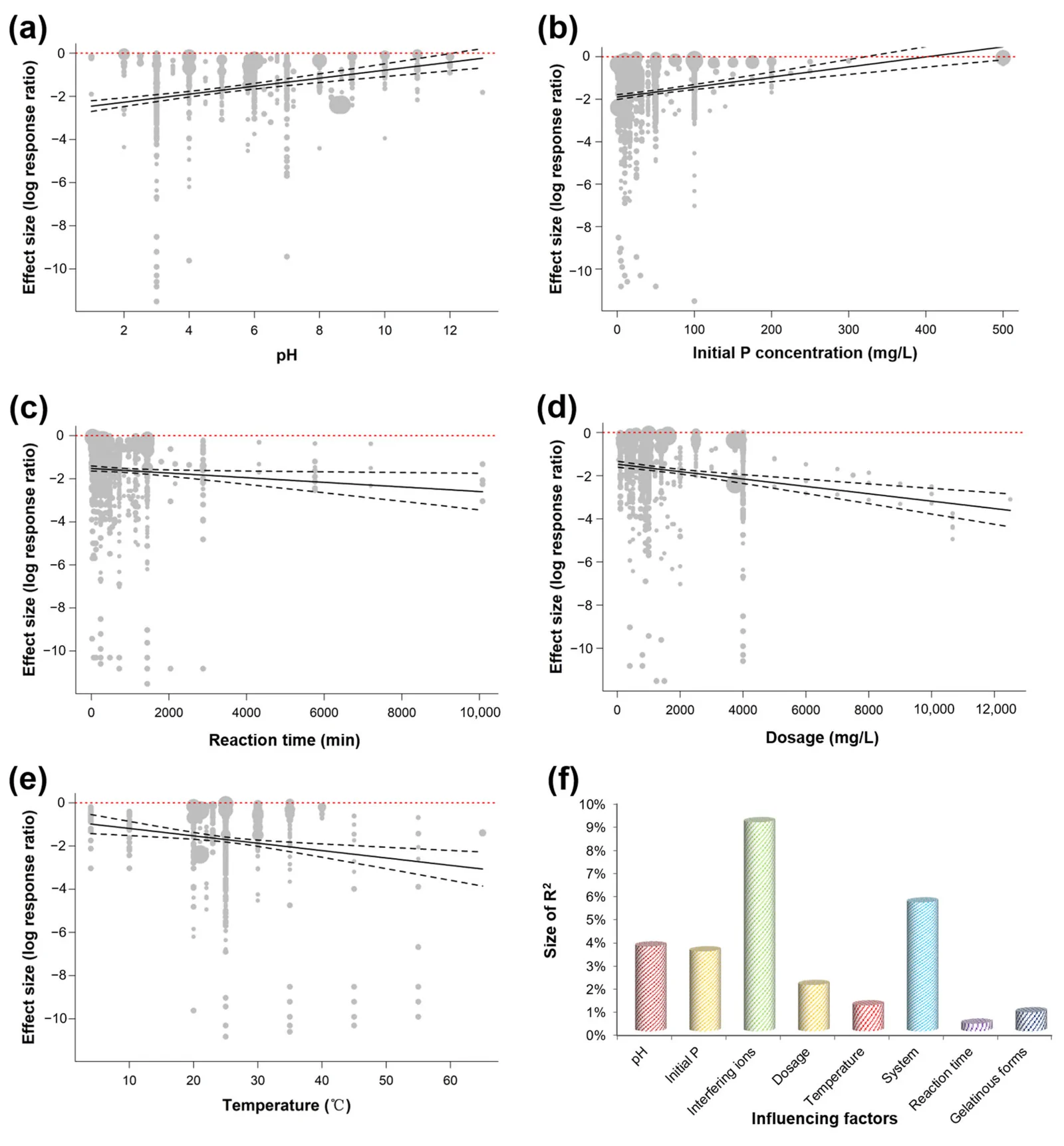
(**a**–**e**) Forest plot of the influence of different water parameters, ambient conditions, and usage parameters and (**f**) a comprehensive comparison of different influencing factors.

## Data Availability

The data that support the findings of this study are available from the corresponding author upon reasonable request.
